# Establishment a real-time reverse transcription PCR based on host biomarkers for the detection of the subclinical cases of *Mycobacterium avium* subsp. *paratuberculosis*

**DOI:** 10.1371/journal.pone.0178336

**Published:** 2017-05-25

**Authors:** Hyun-Eui Park, Hong-Tae Park, Young Hoon Jung, Han Sang Yoo

**Affiliations:** 1 Department of Infectious Disease, College of Veterinary Medicine, Seoul National University, Seoul, Republic of Korea; 2 Department of Animal Resoures Devlopment, National Instiute of Animal Science, Rural Development Administration, Cheonan, Republic of Korea; 3 Institute of Green Bio Science and Technology, Seoul National University, Pyeongchang, Republic of Korea; Cornell University, UNITED STATES

## Abstract

Bovine paratuberculosis (PTB) is a chronic enteric inflammatory disease of ruminants caused by *Mycobacterium avium* subsp. *paratuberculosis* (MAP) that causes large economic losses in the dairy industry. Spread of PTB is mainly provoked by a long subclinical stage during which MAP is shed into the environment with feces; accordingly, detection of subclinical animals is very important to its control. However, current diagnostic methods are not suitable for detection of subclinical animals. Therefore, the current study was conducted to develop a diagnostic method for analysis of the expression of genes of prognostic potential biomarker candidates in the whole blood of cattle naturally infected with MAP. Real-time PCR with nine potential biomarker candidates was developed for the diagnosis of MAP subclinical infection. Animals were divided into four groups based on fecal MAP PCR and serum ELISA. Eight genes (*Timp1*, *Hp*, *Serpine1*, *Tfrc*, *Mmp9*, *Defb1*, *Defb10*, and *S100a8*) were up-regulated in MAP-infected cattle (*p* <0.05). Moreover, ROC analysis revealed that eight genes (*Timp1*, *Hp*, *Serpine1*, *Tfrc*, *Mmp9*, *Defb1*, *Defb10*, and *S100a8*) showed fair diagnostic performance (AUC≥0.8). Four biomarkers (*Timp1*, *S100a8*, *Defb1*, and *Defb10*) showed the highest diagnostic accuracy in the PCR positive and ELISA negative group (PN group) and three biomarkers (*Tfrc*, *Hp*, and *Serpine1*) showed the highest diagnostic accuracy in the PCR negative and ELISA positive group (NP group). Moreover, three biomarkers (*S100a8*, *Hp*, and *Defb10*) were considered the most reliable for the PCR positive and ELISA positive group (PP group). Taken together, our data suggest that real-time PCR based on eight biomarkers (*Timp1*, *Hp*, *Serpine1*, *Tfrc*, *Mmp9*, *Defb1*, *Defb10*, and *S100a8*) might be useful for diagnosis of JD, including subclinical stage cases.

## Introduction

Johne’s disease (JD) is a chronic inflammatory disease of the gastrointestinal tract of ruminants with granulomatous lesions that is caused by *Mycobacterium avium* subsp. *paratuberculosis* (MAP) (Whitlock *et al*., 1996). Johne’s disease can be divided into four stages depending on the clinical signs and MAP shedding levels including the silent, subclinical, clinical, and advanced clinical stage [1]. In the silent stage, the infected animals do not show any clinical sign or excrete MAP into the environment [2]. During the subclinical stage, animals still do not have clinical symptoms; however, they shed low numbers of MAP into environment, which can be circulated in the herd and infect other animals [2]. After subclinical stage, animals enter clinical stage and start to show clinical signs such as gradual weight loss, diarrhea, and decreased milk production [2]. Finally, animals become cachectic and lethargic in advanced clinical stage [2]. Accordingly, it is very important to remove animals in the subclinical stage to control the disease. However, current diagnostic methods are insufficient for diagnosis of subclinical stages of disease [3]. Although fecal culture has been considered a gold standard for the diagnosis of MAP [4], this method is time-consuming and shows low sensitivity, especially in subclinical stages of the disease [5, 6]. PCR allows rapid detection of MAP in clinical samples such as feces, milk and blood [7]; however, PCR-based methods are also limited in their usefulness for diagnosis of subclinical stages of disease because of the low sensitivity [8] and low specificity caused by genetic similarities with other mycobacteria [9, 10]. Although ELISA has been used for detection of antibodies to MAP in clinical samples such as serum and milk, this method is also inadequate for diagnosis of fecal shedders in the subclinical stage, especially in 1–2 year old cattle [11]. Therefore, new diagnostic tools have been requested to detect MAP-infected animals at early stage of infection.

Biomarkers, which are considered indicators of specific pathogenic conditions or therapeutic responses to treatment [12], are commonly used as diagnostic tools for various diseases [13–16]. Recently, host biomarkers discovered using transcriptomics, metabolomics, and proteomics have been proposed as alternative diagnostic methods for paratuberculosis [17–20]. Biomarkers indicating early stages of MAP-infection were proposed by analyzing gene expression profiles of blood in cattle with experimental MAP infection [17, 18]. A metabolic profiling in cattle with experimental infection of MAP revealed that four metabolites (iso-butyrate, branched chain amino acids, leucine, and isoleucine) were increased in serum of the MAP-infected cattle while citrate was decreased [19]. Moreover, six proteins (transferrin, gelsolin isoforms α & β, complement subcomponent C1r, complement component C3, amine oxidase-copper containing 3, and coagulation factor II) were proposed as biomarkers after they were found to increase by at least 2-fold in MAP-infected cattle, as were two proteins (coagulation factor XIII-B polypeptide, and fibrinogen γ chain and its precursor) that were reduced by nearly two-fold in MAP-infected cattle [20]. Our previous studies also proposed several biomarkers that were up-regulated in MAP infected macrophages, mice, and cattle [21–23]. Transcriptional profiles of MAP-infected macrophage RAW 264.7 cells and a mouse model suggested five and three genes as prognostic biomarkers, respectively [21, 22]. β-defensins were also suggested as prognostic biomarkers in subclinical animals of MAP-naturally infected cattle [23]. However, application of those biomarkers for diagnosis of JD has yet to be investigated. Therefore, we developed a real-time PCR method using the biomarkers for diagnosis of bovine paratuberculosis by measuring the gene expression level of several biomarkers in whole blood.

## Materials and methods

### Experimental design and animals

About 300 Holstein cattle were raised on the national farm in Cheonan city which located in mid-west region of the South Korea. The cattle were regularly tested for absence of JD two times per year using fecal PCR and serum ELISA. A total of three to eight year old fourty-four cows were selected for further analysis after detection of MAP-specific antibodies using a commercial ELISA kit (IDEXX Laboratories, Inc., Westbrook, ME, USA) and MAP in the feces by PCR [24]. The detection was performed four times with a 6-month interval to enable accurate classification of infection status. The animals were divided into the following groups based on the results of PCR and ELISA: NN, ELISA and PCR negative; PN, ELISA negative and PCR positive; NP, ELISA positive and PCR negative; PP, ELISA positive and PCR positive. All animal procedures were approved by the National Institute of Animal Science (2013–046). Detailed characteristics of study subjects are shown in Table 1.

**Table 1:** Characteristics of study subjects.

Number of subjects		All (n = 44)	NN group (n = 11)	PN group (n = 12)	NP group (n = 14)	PP group (n = 7)
Heifers, n (%)		44 (100)	11 (100)	12 (100)	14 (100)	7 (100)
Median age (Years)		6 (4 to 9)	4 (4 to 7)	6 (4 to 9)	6.5 (4 to 8)	6 (5 to 8)
Serum ELISA	Positive, n (%)	21 (47.7)	0 (0)	0 (0)	14 (100)	7 (100)
	Negative, n (%)	23 (52.3)	11 (100)	12 (100)	0 (0)	0
Fecal PCR	Positive, n (%)	19 (43.2)	0 (0)	12 (100)	0 (0)	7 (100)
	Negative, n (%)	25 (56.8)	11 (100)	0 (0)	14 (100)	0 (0)

### Selection of biomarker candidates

Nine genes that were significantly up-regulated in MAP infected macrophages, mice, and cattle were selected for use as diagnostic biomarkers based on our previous studies (Table 2) [21–23, 25]. All datasets used in selection of the biomarkers are available at Gene Expression Omnibus (GEO) (http://www.ncbi.nlm.nih.gov/geo website) under accession number GSE62836, http://dx.doi.org/10.4014/jmb.1302.02021, and http://dx.doi.org/10.4014/jmb.1408.08059.

**Table 2:** Mean fold change of selected biomarker genes between infected animals and non-infected animals.

Accession No.	Gene symbol	Gene name	Location	Mean fold change (log2 value)					
				PN vs. NN	P value	NP vs. NN	P value	PP vs. NN	P value
NM_003234.2	*Tfrc*	Transferrin receptor (p90, CD71)	Plasma membrane	1.6	0.0005	1.3	0.0021	1.9	0.0004
NM_174744	*Mmp9*	Matrix metallopeptidase 9	Extracellular space	2.9	0.008	2.4	0.0342	3.6	0.0052
NM_002964.4	*S100a8*	S100 calcium binding protein A8	Cytoplasm	1.6	0.0039	0.9	0.146	1.7	0.0069
NM_002965.3	*S100a9*	S100 calcium binding protein A9	Cytoplasm	0.4	0.6228	0.6	0.2596	1.1	0.0548
NM_174137	*Serpine1*	Serpin peptidase inhibitor	Extracellular space	1.9	0.0041	1.6	0.0183	2.6	0.0009
NM_005143.3	*Hp*	Haptoglobin	Extracellular space	2.3	0.0031	3.2	<0.0001	3.3	0.0003
NM_174471.3	*Timp1*	Tissue inhibitor of metallopeptidase 1	Extracellular space	1.7	<0.0001	1.4	0.0002	0.5	0.415
NM_001324544.1	*Defb1*	Defensin beta 1	Extracellular space	5.2	0.0009	3.3	0.039	1.8	0.5842
NM_001115084.1	*Defb10*	Defensin beta 10	Extracellular space	2.3	0.0017	1.6	0.0313	1.6	0.1009

### Extraction of total RNA from blood

Peripheral blood was collected from the tail vein of cattle using a BD Vacutainer^®^ Plus Plastic K_2_EDTA Tubes. A total of 125 μl of whole blood was then mixed with 125 μl of RNase-free water and 750 μl of Trizol LS reagent (Ambion) and incubated at room temperature for 5 min. Next, 200 μl of chloroform was added to the mixture and it was centrifuged at 13,523 g and 4°C for 15 min. The supernatant was subsequently transferred to an RNAeasy column (Qiagen, Hilden, Germany) and centrifuged at 8,500 g for 15 sec. After washing, RNA was eluted in 30 μl of RNase-free water and immediately stored at -80°C until use.

### Optimization of primer and probe concentrations

The optimal concentration of primer and probe concentration was determined with an identical cDNA template for each biomarker gene. Three concentrations (0.5μM, 0.75μM, 1μM) of both forward and reverse primers with a constant probe concentration were tested. The combination showing the highest fluorescence value was tested at three different concentrations of the probe (0.1μM, 0.2μM, 0.3μM). For further experiment, primer and probe concentration that showing the highest fluorescence value was selected.

### Real-time PCR

Total RNA was employed to prepare cDNA with random primers using a QuantiTect^®^ Reverse Transcription Kit (Qiagen Inc., Valencia, CA, USA) according to the manufacturer’s instructions. The expression of nine biomarker genes was measured by quantitative real time RT-PCR, which was conducted using a Rotor-Gene multiplex PCR kit (Qiagen Inc). In brief, total of 18μl reaction mixture was prepared consists of 10μl Master mix, RNase-free water, 0.5μM forward and reverse primers, and 0.1μM probe for each of the biomarker genes. After that, 2μl of cDNA template was added to a final volume of 20μl. The specificity of the primers and probes for each biomarker genes was confirmed by homology search (https://www.ncbi.nlm.nih.gov/tools/primer-blast) and agarose gel electrophoresis. The primers and probe used in this study are shown in Table 3. Sensitivity of real-time PCR reactions was confirmed by real-time PCR reaction using the known copy numbers calculated from purified PCR products which serially diluted from 10^9^ to 10^2^ copies of the templates. The real-time PCR was conducted for 45 cycles and C_T_ values were obtained. Negative control was included with no template. Real-time PCR was conducted by subjecting the samples to 95°C for 10 min, followed by 50 cycles of 95°C for 15 s and 60°C for 45 s. The expression level was determined by the 2^-ΔΔ^Ct method using the housekeeping gene, β-actin, as a reference.

**Table 3:** Oligonucleotide sequence of primer and probe used for real-time PCR in this study.

Target gene		Primer sequence(5’ to 3’)	PCR product size(base pair)	Reference
*β- actin*	F	GCAAGCAGGAGTACGATGAG	134	In this study
	R	GCCATGCCAATCTCATCTCG		
	Probe	FAM-TTCTAGGCGGACTGTTAGCTGCGTTACAC-BHQ1		
*Mmp9*	F	CCCGGATCAAGGATACAGCC	177	[25]
	R	GGGCGAGGACCATACAGATG		
	Probe	HEX-AGTTTGGCCACGCGCTGGGCTTAGAT-BHQ1		
*Serpine1*	F	CTGCGAAATTCAGGATGCGG	191	[25]
	R	GGGTGAGAAAACCACGTTGC		
	Probe	FAM-AGACTTTGGAGTGAAGGTGTTTCAGCAGG-BHQ1		
*Timp1*	F	TCTGCAACTCCGATGTCGTC	125	In this study
	R	CCTCAAGGCACTGAACCCTT		
	Probe	HEX-GTTCGTGGGGACCGCAGAAGTCAATG-BHQ1		
*Hp*	F	CCAAGTACCAGGACGACACC	131	In this study
	R	ACCATACTCAGCCACAGCAC		
	Probe	FAM-ACGACAAGGAAGACGACACCTGGTATGC-BHQ1		
*S100a8*	F	ATTTTGGGGAGACCTGGTGG	124	[25]
	R	ACGGCGTGGTAATTCCCTTT		
	Probe	FAM-TAACTCCCTGATTGACGTCTACCACAAG-BHQ1		
*S100a9*	F	AGGCTACGGGAAGGGCAG	134	[25]
	R	GCTGGCCTCCTGATTAGTGG		
	Probe	HEX-ATGGAGGTCACGGCCACAGCCAC-BHQ1		
*Tfrc*	F	CAAAGTTTCTGCCAGCCCAC	188	[25]
	R	AACAGAAAGAGACCGCTGGG		
	Probe	HEX-TATCGGGACAGCAACTGGATCAGCAAAG-BHQ1		
*Defb1*	F	CGAATGGAGGCATCTGTTTG	110	In this study
	R	CTTCGCCTTCTTTTACCACGA		
	Probe	FAM-TGCCCTGGACACATGATACAGATTGGCA-BHQ1		
*Defb10*	F	ATCTAAGCTGCTGGGGGAAT	97	In this study
	R	CATTTTACTCGGGGCGCTAA		
	Probe	HEX-GTTTGCTTAACAGGTGCCCTGGAC-BHQ1		

### Statistical analysis

Data are reported as the means ± the standard error of the mean (S.E.M.) of three independent experiments. Statistical significance was determined by ANOVA (p ≤ 0.05) with Dunnett’s post hoc test using the GraphPad Prism software version 7.00 (GraphPad Software, Inc., La Jolla, CA, USA). Receiver operator characteristics (ROC) curve analysis was conducted using the statistical package for social science (SPSS) software version 21.0 (SPSS Inc., Chicago, IL, USA) and the MedCalc Statistical Software version 13.3.3 (MedCalc Software, Ostend, Belgium). Higher AUC scores were considered to show better discriminatory powers as follows: excellent discriminatory power, AUC ≥0.9; good discriminatory power, 0.8≤AUC<0.9; fair discriminatory power, 0.7≤AUC<0.8; poor discriminatory power, AUC<0.7 [26]. The optimal cutoff values were calculated for each ROC curve while maximizing the Youden Index. Sensitivity and specificity were calculated based on cut-off value which showed highest AUC value in the ROC curve for each biomarker gene. A *p* <0.05 was considered to indicate statistical significance.

## Results

### Specificity of probe and primers

**Fig 1:**
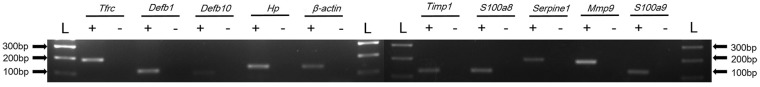
Gel electrophoresis of PCR products of biomarkers genes and β-actin gene. The biomarker genes and β-actin gene expression from bovine whole blood cDNA were confirmed by RT-PCR. A single PCR product was observed with expected size for each biomarker and β-actin gene. No band was observed in the PCR products of negative control without template DNA sample. In the figure (L) indicates 100bp DNA size marker, (+) indicates PCR product with template cDNA, (-) indicates PCR product without cDNA.

Specificity of primers and probes were confirmed by homology search. Also, to confirm the specificity for each biomarker genes, RT-PCR and agarose gel electrophoresis was performed. Single PCR band were confirmed for each biomarker gene and the β-actin gene and non-specific PCR product was not observed confirmed in the negative control with no cDNA sample (Fig 1).

### Sensitivity of real-time PCR reactions

**Fig 2:**
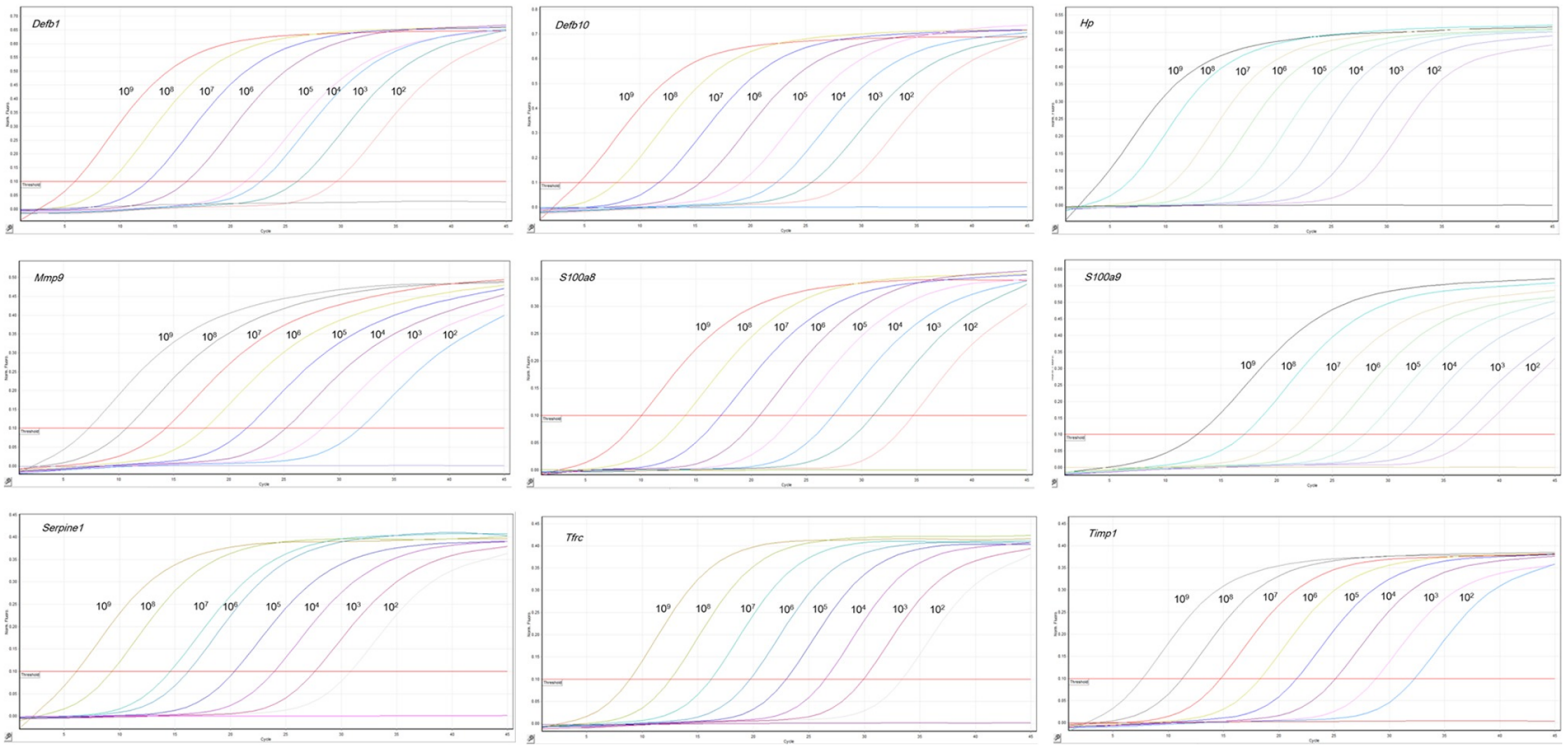
Amplification plots of the biomarker genes in the real-time PCR. Real-time PCR was conducted with PCR product that serially diluted 10-fold from 10^9^ to 10^2^ copy numbers. The emission of fluorescence was measured at each cycle numbers and negative control sample with no template DNA showed no increasing of fluorescence.

Real-time PCR for the each biomarker gene was performed using the specific primers, probes and the purified PCR products. Amplification plots were presented for biomarker genes with increased template copy numbers from 10^2^ to 10^9^. Amplification plot shows that fluorescence increase with increased template copy numbers (Fig 2). Also, real-time PCR was highly sensitive to detect low level of gene expression of biomarker genes (about 10^2^ copies of the template cDNA) and negative control sample with no template DNA showed no increasing of fluorescence (Fig 2).

### Optimization of primer and probe concentrations

The optimal concentration of primer and probe concentration was determined by conducting real-time PCR with three primer and probe concentrations. The combination of forward and reverse primer at 0.5μM for biomarker genes and β-actin gene revealed highest florescence and lowest C_T_ value. With this primer concentration, 0.1μM of probe showed highest florescence and lowest C_T_ value. Combination of 0.5μM forward and reverse primers and 0.1μM probe concentration was used in further analysis.

### Gene expression level of biomarkers in MAP infected cattle

**Fig 3:**
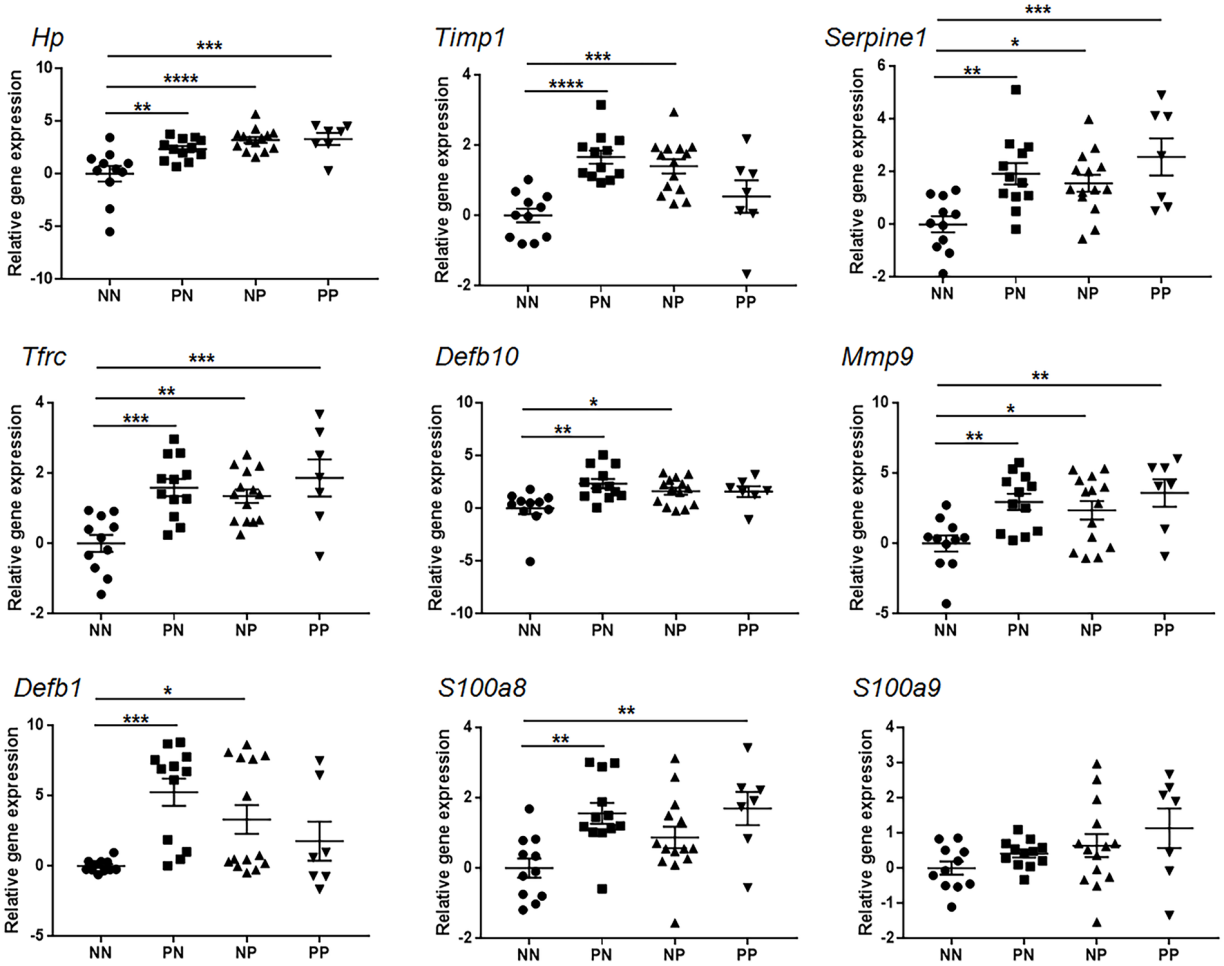
Gene expression level of biomarkers in MAP-infected cattle. The gene expression level of biomarker genes in cattle infected with *Mycobacterium avium* subspecies *paratuberculosis* compared to non-infected cattle. The data are shown as scatter plots with each dot representing a single animal. (*, p<0.05 **, p<0.01 ***, p<0.001 ****, p<0.0001).

Experimental animals were divided into four groups based on the results of fecal PCR and serum ELISA conducted three times with a 6 month interval (Table 1). When compared with the non-infected NN group, expression of eight genes (*S100a8*, *Defb1*, *Defb10*, *Mmp9*, *Timp1*, *Hp*, *Serpine1*, and *Tfrc*) showed higher expression in the PN group (p<0.05), while higher expression of seven other genes (*Timp1*, *Hp*, *Serpine1*, *Tfrc*, *Defb1*, *Defb10*, and *Mmp9*) was observed in the NP group (*p* <0.05). Moreover, in the PP group, five genes (*S100a8*, *Mmp9*, *Hp*, *Serpine1*, and *Tfrc*) showed significantly higher expression in the PP group (*p* <0.05). Four genes (*Tfrc*, *Hp*, *Serpine1*, and *Mmp9*) were up-regulated in all infected groups, while three genes (*Timp1*, *Defb1*, and *Defb10*) were up-regulated in the PN group and the NP group, and *S100a8* was up-regulated in the PN group and the PP group (Fig 3). The mean fold changes of each biomarker are shown in Table 2.

### Discrimination between infected and non-infected animals

**Fig 4:**
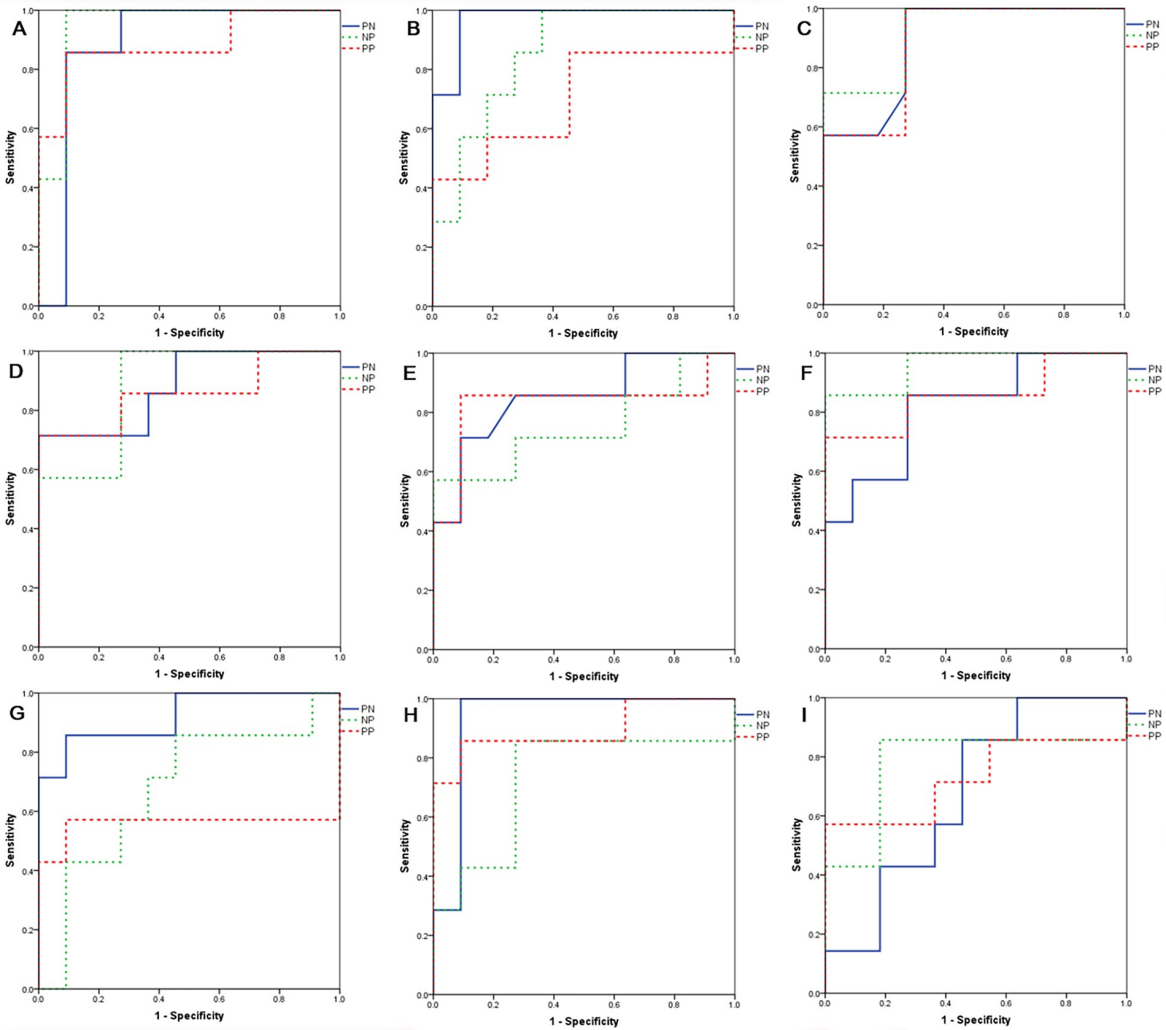
Discriminatory ability of biomarkers between infected animals and control animals. Receiver operator characteristics curves of biomarker genes in cattle infected with *Mycobacterium avium* subspecies *paratuberculosis* compared to non-infected cattle. (A) *Hp*; (B) *Timp1*; (C) *Serpine1*; (D) *TFRC*; (E) *Defb10*; (F) *Mmp9*; (G) *Defb1*; (H) *S100a8*; (I) *S100a9*

The AUC score of biomarkers was calculated during ROC analysis. In the PN group, the AUC scores of eight genes (*Timp1*, *Defb1*, *Tfrc*, *Defb10*, *S100a8*, *Serpine1*, *Mmp9*, and *Hp*) were ≥0.8. In the NP group, four genes (*Hp*, *Timp1*, *Tfrc*, and *Serpine1*) had AUC scores ≥0.8, while six genes (*S100a8*, *Hp*, *Serpine1*, *Tfrc*, *Mmp9*, and *Defb10*) in the PP group had AUC scores ≥0.8 (Fig 4). When the diagnostic accuracies of individual biomarkers were calculated by ROC curve analysis, the most accurate biomarker in the PN group was *Timp1*, with an AUC value of 0.985, while the most accurate biomarker in the NP group was *Hp*, with an AUC value of 0.942. Additionally, the most accurate biomarker in the PP group was *S100a8*, with an AUC value of 0.896. Similarly, in the PN group, *Timp1* showed the most accurate diagnostic performance, with a sensitivity of 100% and a specificity of 90.9%. In the NP group, *Hp* showed the most accurate diagnostic performance, with a sensitivity of 92.9% and a specificity of 90.9%. Moreover, *S100a8* showed the most accurate diagnostic performance in the PP group, with a sensitivity of 85.7% and a specificity of 90%. Other details pertaining to the diagnostic performance of biomarkers are shown in Table 4.

**Table 4:** Diagnostic performance of biomarkers for diagnosis of JD.

Biomarker	AUC			P value			Cut-off (fold change)			Sensitivity (%)			Specificity (%)		
	PN vs. NN	NP vs. NN	PP vs. NN	PN vs. NN	NP vs. NN	PP vs. NN	PN vs. NN	NP vs. NN	PP vs. NN	PN vs. NN	NP vs. NN	PP vs. NN	PN vs. NN	NP vs. NN	PP vs. NN
*Tfrc*	0.909	0.89	0.857	<0.0001	<0.0001	0.0012	>0.94	>0.47	>0.94	75	92.9	71.4	100	72.7	100
*Mmp9*	0.871	0.76	0.857	<0.0001	0.0102	0.0012	>0.455	>2.73	>2.73	91.7	57.1	71.4	72.7	100	100
*S100a8*	0.894	0.721	0.896	<0.0001	0.045	<0.0001	>0.829	>-0.095	>0.829	91.7	92.9	85.7	90.9	54.5	90
*S100a9*	0.693	0.669	0.727	0.11	0.1315	0.1365	>-0.07	>0.511	>0.856	91.7	57.1	57.1	54.5	81.8	100
*Serpine1*	0.875	0.864	0.883	<0.0001	<0.0001	<0.0001	>0.463	>1.022	>0.372	91.7	78.6	100	72.7	100	72.7
*Hp*	0.864	0.942	0.883	<0.0001	<0.0001	0.0001	>1.806	>1.806	>1.806	75	92.9	85.7	90.9	90.9	90.9
*Timp1*	0.985	0.929	0.701	<0.0001	<0.0001	0.1815	>0.682	>0.682	>1.022	100	78.6	42.9	90.9	90.9	100
*Defb1*	0.955	0.786	0.558	<0.0001	0.0024	0.7681	>0.951	>0.325	>0.325	83.3	64.3	57.1	100	90.9	90.9
*Defb10*	0.905	0.773	0.831	<0.0001	0.0052	0.0118	>1.154	>1.815	>1.154	83.3	57.1	85.7	90.9	100	90.9

## Discussion

Early diagnosis of JD is the most important requirement to eradicate it from MAP-infected herds. However, current diagnostic methods are not sufficient for the diagnosis of subclinical stage animals that are actively dispersing MAP into the environment via fecal shedding [2]. Recently, several studies have attempted to diagnose subclinical stages of JD by analyzing host-pathogen interactions, including gene expression, miRNA, protein, and metabolites to MAP infection [17–20, 27]. Some of the studies have been conducted to identify prognostic biomarkers of JD by understanding host response to infection during the progression of JD [21, 28–31]. However, no attempt has been made to apply biomarkers as diagnostic tools. Therefore, the present study was conducted to diagnose MAP infection using a real-time PCR method based on potential prognostic biomarkers.

In the present study, several biomarkers showed good discriminatory ability (AUC≥0.8) between MAP-infected cattle and non-infected cattle. Three genes (*Hp*, *Serpine1*, and *Tfrc*) showed good discriminatory ability (AUC≥0.8) in fecal PCR-positive and/or serum ELISA-positive groups (PN, NP, PP). Acute phase proteins are blood proteins that respond to infection and inflammation and have been used as diagnostic and prognostic biomarkers in veterinary medicine [32]. *Hp* is the major acute phase protein of cattle that responds to infection [33, 34]. Moreover, *Hp* is known to exert anti-inflammatory activity by down-regulating neutrophil activity via inhibition of both lipoxygenase and cycloxygenase [35] and to inhibit bacterial growth by interfering with iron acquisition by the host cell [36]. Moreover, *Hp* inhibits phagocytosis and intracellular killing of pathogens [37]. This anti-inflammatory response induced by *Hp* might reduce the harmful aspects of inflammation that could be destructive to the host itself. In that regard, up-regulation of *Hp* in MAP-infected animals might be a host response to early infection of MAP. *Hp* showed highest diagnostic accuracy for the NP group and whole infected animals, with AUC values of 0.942 and 0.901, respectively.

The initial response to MAP infection is dominant cell-mediated immunity, which is characterized by increasing interferon gamma release [38]. *Serpine1* is known to be an essential element of the fibrinolytic system that is related to blood coagulation [39]. *Serpine1* also acts as an inflammatory mediator by increasing the level of interferon gamma in blood to eliminate the pathogen in the early phase of an infectious disease [40, 41]. Therefore, increasing gene expression levels of *Serpine1* might be related to interferon gamma release due to MAP infection. In addition, expression of MAP0403 in MAP was increased in infected macrophages and MAC-T cells in recent study [42]. MAP0403 is kind of serine protease which served as a key element of the stress response network in intraphagosomal survival of MAP [42]. Up-regulation of *Serpine1* might be a counter response to intraphagosomal survival of MAP in host cells. The diagnostic accuracy of *Serpine1* was good (AUC≥0.8) in all infected animals (PN, NP, PP group).

Iron is an important nutrient in innate immune response to bacterial pathogen [43]. *Tfrc*, which is one of the key elements of iron metabolism, transfers iron to cells from transferrin protein [43]. *Tfrc* is known to down-regulated in response to intracellular pathogen infection; however, its expression was significantly increased in all infected animals in the present study. This phenomenon might be related to the alternative iron acquisition system of MAP, which acts in a host-independent manner using mycobactin [44]; however, further studies are needed to confirm this.

*Mmp9* is a matrix metalloproteinase related to leukocyte migration to infection sites and tissue destruction if it is secreted in excess amounts [45]. The level of *Mmp9* was regulated by *Timp1*, which inhibits the activity of MMP9 [45]. *Mmp9* and *Timp1* are known to be up-regulated in tuberculosis infection and have therefore been proposed as biomarkers for diagnosis of tuberculosis [46]. The simultaneous up-regulation of *Mmp9* and *Timp1* in infected animals might be caused by inflammatory conditions due to the early stages of MAP infection. Two genes (*Mmp9* and *Timp1*) showed good discriminatory ability (AUC≥0.8) in the PN group.

β-defensins exhibit antimicrobial functions, providing first protection against pathogens while playing an immune-modulation role [47]. Moreover, β-defensins interplay between innate and adaptive immune responses by down-regulating pro-inflammatory cytokines [48]. In the present study, *Defb1* and *Defb10* were significantly up-regulated in both the PN group and the NP group. Moreover, *Defb1* and *Defb10* showed excellent discriminatory ability (AUC≥0.9) in the PN group.

*S100a8* and *S100a9* are members of a calcium-binding cytosolic protein family that are located in the cytoplasm [49]. *S100a8* and *S100a9* form a heterodimer known as calprotectin that induces an inflammatory response via activation of TLR4 signaling [50]. Moreover, calprotectin is known to induce leukocyte migration in the early phase of bacterial infection [51]. In previous studies, serum S100A8/A9 have been proposed as prognostic biomarkers for disease progression and therapeutic response in inflammatory bowel diseases (IBD)[52, 53]. In the present study, *S100a8* showed good discriminatory ability (AUC≥0.8) in the PN and PP groups. However, gene expression of *S100a9* was not significant in all infected animals. Generally, *S100a8* and *S100a9* exist as heterodimers, but they also exist as homodimers [54]. The inconsistent gene expression levels between *S100a8* and *S100a9* might be related to the presence of the homodimer form.

An ideal biomarker for diagnosis of JD should be able to discriminate between infected and non-infected animals with high sensitivity and specificity. Our data showed that the response of eight biomarkers (*Hp*, *Timp1*, *Mmp9*, *Serpine1*, *Tfrc*, *S100a8*, *Defb1*, and *Defb10*) significantly discriminated MAP-infected and non-infected animals. Moreover, eight biomarkers (*Hp*, *Timp1*, *Mmp9*, *Serpine1*, *Tfrc*, *S100a8*, *Defb1*, and *Defb10*) showed good accuracy (AUC≥0.7) for diagnosis of subclinical animals. Additionally, four genes (*Timp1*, *S100a8*, *Defb1*, and *Defb10*) showed sensitivity over 80% and specificity over 90%. It is generally very difficult to detect subclinical stages of JD using currently available diagnostic methods such as bacterial culture, fecal PCR and serum ELISA [3]. Fecal PCR is a reliable method for diagnosis of MAP infection; however, intermittent shedding of MAP into feces because of immunological changes during the progress of disease can interfere with accurate diagnosis [55]. Moreover, although serum ELISA is a simple, fast and cost-effective method for diagnosis of JD, it is known to have low sensitivity for MAP-infected animals that do not show clinical signs [56]. However, our real-time PCR method based on biomarkers showed relatively precise diagnostic results. In that regard, combination of eight biomarker genes (*Hp*, *Timp1*, *Mmp9*, *Serpine1*, *Tfrc*, *S100a8*, *Defb1*, and *Defb10*) might be used for diagnosis of JD, including in subclinical stage animals.

In conclusion, a real-time PCR method was developed based on eight biomarkers that can be used as a new diagnostic tool for JD with good diagnostic performance. Moreover, this real-time PCR based on biomarkers might be used for diagnosis of JD, especially in subclinical stage animals that cannot be detected by current diagnostic methods. Although our developed diagnostic method might be applied to field test, this method will be more concreted if possible limitations in our study such as the low number of samples and sampling times would be addressed in future studies by including large scale field investigations.
